# Brachytherapy for targeting the immune system in cervical cancer patients

**DOI:** 10.1186/s12865-023-00559-y

**Published:** 2023-08-09

**Authors:** Isabel Linares, Miguel Ángel Berenguer Frances, Rut Cañas, Dina Najjari, Cristina Gutiérrez, Susanna Marín, Silvia Comas, Ferran Guedea, Monica Pujol

**Affiliations:** 1 Radiation Oncology Department, Hospital Duran i Reynals, Institut Català d’Oncologia (ICO), Avinguda de la Gran Via de l’Hospitalet 199-203, L’Hospitalet de Llobregat, 08098 Barcelona, Spain; 2 Radiobiology and Cancer Group, ONCOBELL Program, Institut d’Investigació Biomèdica de Bellvitge (IDIBELL), Avinguda de la Gran Via de l’Hospitalet 199-203, L’Hospitalet de Llobregat, 08098 Barcelona, Spain; 3 Radiation Oncology Department, Hospital La Fe, Avinguda de Fernando Abril Martorell 106, 46026 Valencia, Spain; 4 Radiation Oncology Department, Hospital Germans Trias i Pujol, Institut Català d’Oncologia (ICO), Carretera de Canyet, s/n, 08916 Badalona, Spain; 5 Unitat d’Antropologia Biològica, Departament de Biologia Animal, Biologia Vegetal i Ecologia, Universitat Autònoma de Barcelona, 08193 Bellaterra, Barcelona, Spain

**Keywords:** Immune system, Cervical cancer, Brachytherapy, Flow cytometry, Natural killer cells, Immunosuppressive cells

## Abstract

**Background:**

New combinations based on standard therapeutic modalities and immunotherapy require understanding the immunomodulatory properties of traditional treatments. The objective was to evaluate the impact of brachytherapy (BT) on the immune system of cervical cancer and to identify the best modality, High-dose-rate brachytherapy (HDR-BT) vs. Pulsed-dose-rate (PDR-BT), to target it.

**Methods:**

Nineteen patients enrolled in a prospective study received chemoradiation (CRT) and subsequently HDR-BT or PDR-BT. Peripheral blood samples were obtained for immunophenotyping analysis by flow-cytometry before CRT, BT, and two and four weeks after BT. The Friedman one-way ANOVA, Conover post hoc test, and the Wilcoxon signed-rank test were used to compare changes in cell populations at different periods, perform multiple pairwise comparisons and assess differences between treatment groups (PDR and HDR).

**Results:**

Natural killer cells (NKs) were the best target for BT. Patients receiving HDR-BT achieved significantly higher values ​​and longer time of the CD56dimCD16 + NK cells with greater cytotoxic capacity than the PDR-BT group, which presented their highest elevation of CD56-CD16 + NK cells. Furthermore, both BT modalities were associated with an increase in myeloid-derived suppressor cells (MDSCs), related to a worse clinical prognosis. However, there was a decrease in the percentage of CD4 + CD25 + Foxp3 + CD45RA + regulatory T cells (Tregs) in patients receiving HDR-BT, although there were no significant differences between BT.

**Conclusions:**

Immune biomarkers are important predictive determinants in cervical cancer. Higher cytotoxic NK cells and a trend toward lower values of Tregs might support the use of HDR-BT to the detriment of PDR-BT and help develop effective combinations with immunotherapy.

**Supplementary Information:**

The online version contains supplementary material available at 10.1186/s12865-023-00559-y.

## Introduction

Cervical cancer is among the most common malignancies among female patients worldwide [1]. In most cases, high-risk subtypes of the human papillomavirus (HPV) persistent infections cause the disease [2, 3]. Due to its viral etiology, helper T cells (CD4+), cytotoxic T cells (CD8+), and Natural Killer cells (NKs) play an essential role in papillomavirus elimination and disease control [3, 4]. For this reason, cervical cancer can become a target for immunotherapy treatments [5]. Nevertheless, cervical cancer cells can evade the immune system and promote tumor progression by inhibiting antitumoral immunity [6, 7]. Several studies have observed high expression rates of molecules involved in the down-regulation of T-cell activation, Cytotoxic T-lymphocyte antigen-4 (CTLA-4) or Programmed cell death-1/programmed cell death-ligand 1 (PD-1/PD-L1), as well as an expansion of different immunosuppressor cells, as regulatory T cells (Tregs), macrophages associated to tumors and Myeloid-derived suppressor cells (MDSCs) in cervical cancer and their association with malignant transformation and progression [8, 9].

The current standard of care for locally advanced cervical cancer is definitive chemoradiation (CRT), incorporating concurrent weekly or triweekly cisplatin with external beam radiation therapy (EBRT) followed by high-dose-rate (HDR) or pulse-dose-rate (PDR) brachytherapy (BT) boost [10]. However, the rate of response to treatment is highly variable, and the mechanisms that underlie this heterogeneity in response rates are not well understood [11]. A better understanding of immunotherapy and clinical reports about standard/abscopal effects has a crucial role in radiation-induced antitumor immunity [12]. There is evidence that radiotherapy (RT) induces distinct tumor cell death forms and, consequently, the release of pro-inflammatory cytokines, chemokines, tumor antigens, and other danger signals. Radiation may promote a large amount of tumoral neoantigens that are then presented to the T lymphocytes. Therefore, radiation carries the potential to initiate the adaptive and innate immune responses, resulting in systemic antitumorigenic effects inside and outside of the irradiation field. However, by increasing immunosuppressive cells, RT can inhibit antitumoral immunity [13–15]. Some studies reveal that conventional fractionated RT increases MDSCs and Tregs. Both cell types are the most resistant to radiation [16]. However, there is evidence that RT can reduce both cell types when ablative doses in hypofractionated schemes instead of multiple fractions at lower doses are applied [17–20].

Studies of immunomodulation produced by radiation in cervical cancers are based on animal models. Clinical studies that include different timepoints are limited or are focused on immune responses in normofractionated schemes. As a result, little is known about immune activation kinetics during BT. Like other hypofractionated RT treatments, BT has a great potential to stimulate the immune system. HDR is widely used instead of LDR and has substantial advantages (dose optimization, radiation safety, and short treatment time). LDR is considered radiobiologically advantageous over HDR in terms of late tissue effects, although not reflected in randomized trials reporting that probabilities of local control (LC) and overall survival (OS) were similar for LDR and HDR treatments [21]. We hypothesized that radiation doses delivered with HDR and PDR brachytherapy in patients with cervical cancer could trigger immune stimulation, especially in a shorter treatment time (HDR). For this reason, the main goal was to evaluate changes in the different immune cell populations in peripheral blood after HDR-BT and PDR-BT and to identify the most effective radiation technique to target the immune system.

## Methods

### Patients

Participants of the current study signed previous informed consent (protocol code HDR-01, reference PR 245/15) approved by the Ethical committee of clinical research from the hospital before they were recruited. From June 2016 to 2018, 19 patients were included with histological confirmation of cervical cancer with radiotherapy intended curative treatment. Patients with the following criteria were not included in the study: hysterectomy before RT, malignancy disease except for non-melanoma skin cancer, history of pelvic RT, and patients with a current state of immunosuppression caused by immunosuppressive drugs or systemic disorders.

### Treatment

All study participants received EBRT (Daily fractions of 1.8-2 Gy up to 45–50 Gy) concurrent with 40 mg/week of cisplatin. After this, patients received HDR or PDR brachytherapy depending on equipment availability. For HDR-BT, Ir-192 was used as a radioisotope. The prescribed dose was 28 Gy applied in 4 sessions of 7 Gy per fraction (EQD2 equivalent to 85–90 Gy in the tumor), applied by using two implants and two fractions for each implant, each fraction separated by at least 6 h. For PDR-BT, Ir-192 was used as radioisotope in a prescribed dose of 35 Gy, 0.8 Gy/pulse in a single application, with 3–4 hospitalization days. Treatment was planned by Oncentra system (Elekta AB, Veenendaal, The Netherlands).

### Blood samples

Peripheral blood samples (9 mL) were drawn in ethylenediaminetetraacetic acid (EDTA) and obtained before CTRT, BT, and two and four weeks after BT treatment. Fresh blood was used, and the samples were processed within 24 h, so that the cell viability procedure could be omitted.

Peripheral blood mononuclear cells (PBMCs) were isolated from a heparinized venous blood sample by density gradient centrifugation. The blood was diluted 1:1 with saline before being layered onto Ficoll® Plaque Plus (GE Healthcare Bio-Sciences, Pittsburgh, PA, USA). After centrifugation, PBMCs were collected from the plasma-Ficoll interphase and used for flow cytometry assays.

### Flow cytometry

All 50–100 uL of blood was added to the appropriate tubes, and cells were processed according to the manufacturer´s instructions. The antibody panels used were the following:

- Lymphocyte Phenotyping panel (DuraCloneTM, Beckman Coulter Life Sciences, Indianapolis, IN, USA): CD16, CD56, CD19, CD14, CD4, CD8, CD3, and CD45 antibodies.
- Regulatory T Cells panel (DuraCloneTM, Beckman Coulter Life Sciences): CD45RA, CD25, CD39, CD3, CD45, CD4, Helios and intracellular Foxp3 antibodies.
- Myeloid-derived Suppressor Cells (MDSC) panel (DuraCloneTM, Beckman Coulter Life Sciences): CD45, HLA-DR, CD14, CD33, and CD11b antibodies.

All flow cytometry data were acquired on a 10-color/3-laser Gallios flow cytometer (Beckman Coulter). The instrument has not been altered. The stability of the flow cytometer was assured through a quality control procedure using Flow-Check Pro Fluorospheres (Beckman Coulter). According to the manufacturer’s instructions, a compensation matrix for each panel was created using the compensation tubes supplied with each panel. A minimum of 100,000 leucocytes for lymphocyte phenotyping, 100,000 leucocytes for MDSC analyses, and 40,000 leucocytes for regulatory T cells analysis were established [22]. Data were manually analyzed using FlowJo software v. 10.5 (Tree Star Inc., Ashland, OR, USA).

### Statistical analysis

Categorical data were expressed as frequencies and percentages, and continuous data as median and range. The Friedman one-way ANOVA was used to compare changes in cell populations at different periods. Conover test post hoc to perform multiple pairwise comparisons; subsequently, p-values adjustment was performed by False Discovery Rate (FDR). The Wilcoxon signed-rank test was used to assess the cell populations’ differences between both treatment groups (PDR and HDR). Statistical significance was set at p < 0.05. Data were analyzed using the R statistical program (version 3.5.0).

## Results

All 19 patients were diagnosed with locally advanced cervical cancer (IBI-IIIB). The age range was 40 to 74 years, and the median age was 55.9 years. Seven patients were treated with HDR-BT and 12 with PDR-BT. In Table 1, the clinical characteristics of the patients can be seen. Four relapses were observed (3 in the PDR arm and 1 in the HDR arm), and after a median follow-up of 47 months, at 3-year OS was 89% for both groups, with 2 deaths in the PDR arm. No significant differences were found between both treatment groups in LC and OS.

**Table 1:** Patient characteristics

	Number (%)
Number of patients	19 (100)
Age, years [mean(range)]	55.9 (40–74)
FIGO stage ^1^	
IB1	1 (5.3)
IB2	1 (5.3)
IIA	3 (15.8)
IIB	7 (36.8)
IIIA	3 (14.8)
IIIB	4 (21.0)
Histopathology	
Squamous cell carcinoma	17 (89.5)
Adenocarcinoma	1 (5.3)
Mucinous carcinoma	1 (5.3)
Tumor differentiation grade	
Well-differentiated	3 (15.8)
Moderately differentiated	10 (52.6)
Poorly differentiated	6 (31.6)
Human papillomavirus (HPV) infection	19 (100)
External beam radiation therapy	19 (100)
Brachytherapy	
HDR	7 (36.8)
PDR	12 (63.2)
Platinum-based chemotherapy	
Yes	18 (94.7)
No	1 (5.3)
Lymphadenectomy	
Yes	7 (36.8)
No	12 (63.2)

^1^ FIGO, International Federation of Gynecology and Obstetrics

### Lymphocytes subpopulations

Lymphocytes were identified from CD45. T lymphocytes were distinguished from total lymphocytes using the characteristic CD3 marker. From CD3 cells, CD4 + helper lymphocytes were separated from CD8 + cytotoxic. B lymphocytes were identified by characterization CD3-CD19+. A negative selection of the CD3-CD19-CD20- markers was made to select the NK cells; subsequently, through the combination of CD56 and CD16, subpopulations of NK cells were obtained.

When Friedman´s one-way ANOVA test was applied to compare changes in cell populations at different periods, no significant differences were observed in the lymphocyte populations.

When different subpopulations of lymphocytes were analyzed separately, there were no significant differences when comparing different periods in almost all populations and for both treatments (HDR and PDR). A summary of subpopulations of lymphocyte values can be seen in Table 2, and Additional File.

**Table 2:** Difference of lymphocyte subpopulation percentages between baseline and after completion of CT-RT and two and four weeks after completion HDR and PDR Brachytherapy

	Baseline vs. Treatment Friedman Test	CT-RT vs. Baseline Conover Test	2w BT vs. Baseline Conover Test	4w BT vs. Baseline Conover Test	2w BT vs. End CT-RT Conover Test	4w BT vs. End CT-RT Conover Test	2w vs. 4w after BT Conover Test
	p	p	p	p	p	p	p
***HDR***							
Total lymphocytes	< 0.1558	< 0.0566	< 0.7776	< 0.6015	< 0.0566	< 0.1200	< 0.6881
B-cells CD19^+^	< 0.0083	< 0.0020	< 0.0002	< 0.1729	< 0.1729	< 0.0346	< 0.0020
Total T-cells CD3^+^	< 0.0342	< 0.0105	< 0.7548	< 0.0158	< 0.0105	< 0.7548	< 0.0105
T-cells CD3^+^CD4^+^	< 0.0006	< 0.6388	< 2.9E^-^05	< 3.4E^-^06	< 1.4E^-^05	< 2.8E^-^06	< 0.2031
T-cells CD3^+^CD8^+^	< 0.0006	< 0.6356	< 3.8E^-^06	< 8.1E^-^06	< 4.7E^-^06	< 1.6E^-^05	< 0.4174
CD4^+^/CD8^+^ ratio	< 0.0007	< 0.6470	< 7.9E^-^06	< 7.9E^-^06	< 1.5E^-^05	< 7.9E^-^06	< 0.6470
NK CD56^dim^CD16^+^	< 0.0144	< 0.0009	< 0.6082	< 0.6082	< 0.0014	< 0.0014	1
NK CD56^high^CD16^+^	< 0.0197	< 0.0474	< 0.0077	< 0.0008	< 0.2592	< 0.0449	< 0.2396
NK CD56^dim^CD16^-^	< 0.0144	< 0.0009	< 0.6082	< 0.6082	< 0.0014	< 0.0014	1
NK CD56^-^CD16^+^	< 0.0002	< 5.8E^-^08	< 6.9E^-^08	< 0.0013	< 0.5875	< 1.9E^-^05	< 4.5E^-^05
***PDR***							
Total lymphocytes	< 0.2724	< 0.2683	< 0.4990	< 0.0141	0.5864	0.1263	0.0563
B-cells CD19^+^	< 6.4E^-^06	< 5.3E-12	< 2.3E^-^13	< 2.4E^-^07	0.1028	0.0002	2.1E^-^06
Total T-cells CD3^+^	< 0.0828	< 0.0003	< 0.0152	< 0.0208	0.1504	0.1080	0.7767
T-cells CD3^+^CD4^+^	< 0.0077	< 0.7607	< 0.0012	< 2.9E^-^05	0.0006	2.3E^-^05	0.1612
T-cells CD3^+^CD8^+^	< 0.0040	< 0.5344	< 8.6E^-^05	< 6.4E^-^06	0.0004	2.05E^-^05	0.2616
CD4^+^/CD8^+^ ratio	< 0.0006	1	< 2.5E^-^05	< 1.3E^-^07	2.5E^-^05	1.3E^-^07	0.0629
NK CD56^dim^CD16^+^	< 0.0005	< 3.2E-09	< 2.1E^-^06	< 6.3E^-^06	0.0190	0.0067	0.6143
NK CD56^high^CD16^+^	< 0.0404	< 0.0300	< 4.8E^-^05	< 0.0300	0.0120	1	0.0120
NK CD56^dim^CD16^-^	< 0.0019	< 8.0–07	< 2.5E^-^07	< 0.0001	0.5242	0.0745	0.0220
NK CD56^-^CD16^+^	< 0.0024	< 2.5E-05	< 1.6E^-^07	< 9.0E^-^06	0.0673	0.6336	0.1419

**Fig. 1:**
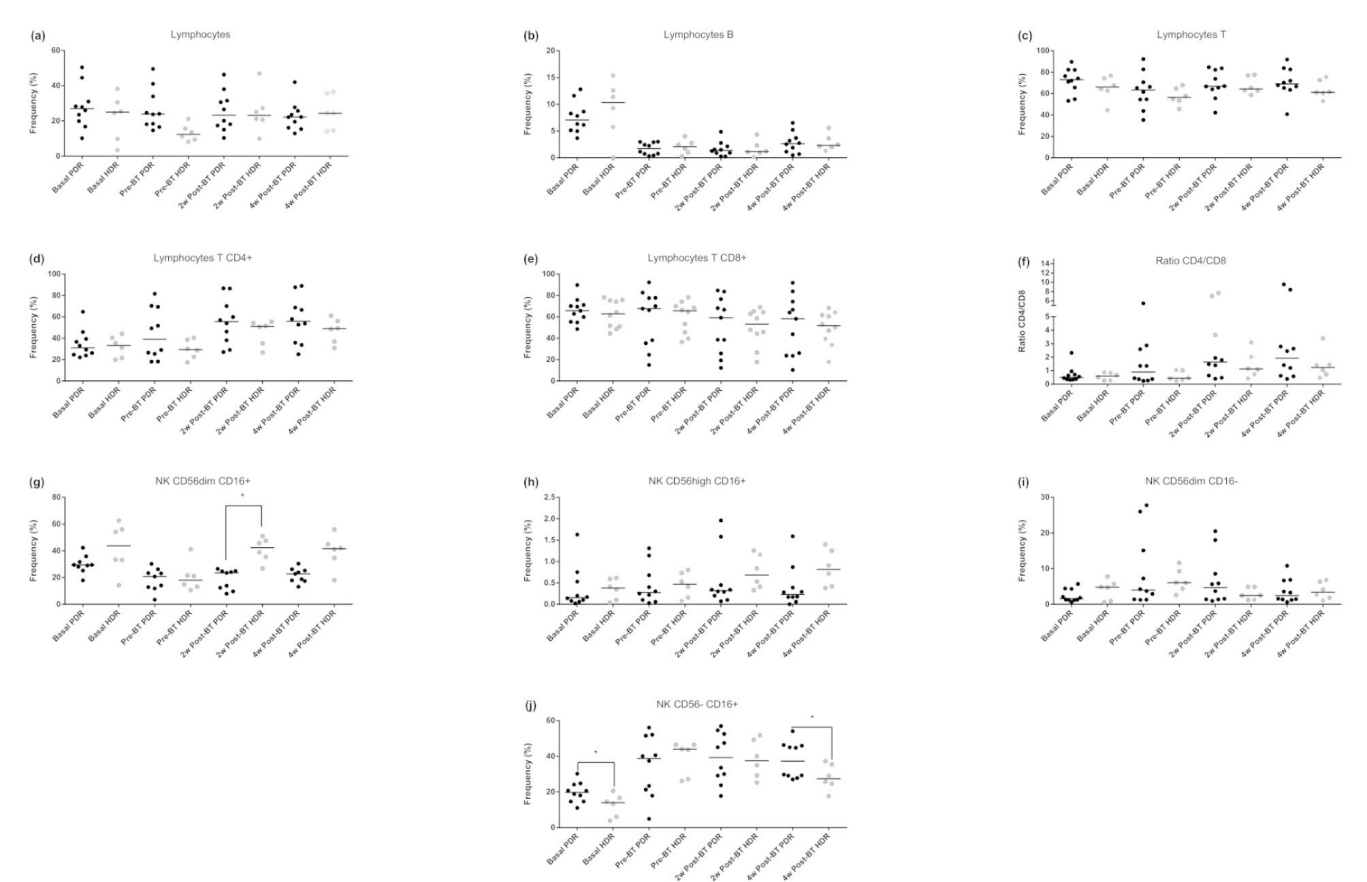
Comparison of median values of cell populations during the study period at different time points between HDR and PDR brachytherapy groups: (**a**) Lymphocytes population; (**b**) Lymphocytes B population; (**c**) Lymphocytes T population; (**d**) Lymphocytes T CD4 + subpopulation; (**e**) Lymphocytes T CD8 + subpopulation; (f) Ratio CD4/CD8; (**g**) NK CD56dimCD16 + subpopulation; (**h**) NK CD56highCD16 + subpopulation; (**i**) NK CD56dimCD16- subpopulation; (**j**) NK CD56-CD16 + subpopulation. * A significant increase in CD56dimCD16 + NK cells in HDR-BT compared to the PDR group at 2w post-BT is shown in Figure (**g**). While an increase in the percentage of the CD56-CD16 + NK cells in the PDR-BT group at 4w post-BT is observed in Figure (**j**)

When both BT techniques (HDR and PDR) were compared using the Wilcoxon signed-rank test, it did not detect significant differences in the B lymphocyte populations, total T lymphocytes, or their CD4 + and CD8 + subpopulations (Fig. 1).

Significant differences were observed for some NK cell subpopulations when both BT techniques were compared using the Wilcoxon signed-rank test. (Fig. 1). An increase in CD56dimCD16 + NK cells was observed in patients treated with HDR-BT compared to the PDR group. At the same time, a significant increase in the percentage of the CD56-CD16 + NK cells was detected in the PDR-BT group.

### Regulatory T cells (Tregs)

The strategy used to analyze different phenotypic and functional Treg subsets was screening lymphocytes according to side scattering and forward scattering characteristics, which were also blocked for CD4 + T cells. Then, CD4 + T cells were examined for CD4 + CD25 + FoxP3 + Helios + cell populations. Treg cells were also divided into functional subsets based on CD45RA and FoxP3 expression.

Both Tregs subpopulations and groups analyzed showed an increase in the different endpoints compared with the baseline. Significant differences were observed when the different endpoints were compared in both subtypes in HDR patients (Friedman one-way ANOVA p = 0.0061 for CD4 + CD25 + FoxP3 + CD45RA + and p = 0.0293 for CD4 + CD25 + FoxP3 + CD45RA- subpopulations). In PDR patients, we found significant differences only in CD4 + CD25 + FoxP3 + CD45RA- subpopulation (Friedman one-way ANOVA p = 0.0384).

The CD4 + CD25 + FoxP3 + CD45RA + subpopulation of cells had a similar pattern in both groups (HDR and PDR) when each endpoint was compared with the baseline. A summary of Tregs values can be seen in Table 3, and Additional File.

**Table 3:** Difference of Regulatory T cells subpopulation percentages between baseline and after completion of CRT and two and four weeks after completion HDR and PDR Brachytherapy

	Baseline vs. Treatment Friedman Test	CT-RT vs. Baseline Conover Test	2w BT vs. Baseline Conover Test	4w BT vs. Baseline Conover Test	2w BT vs. End CT-RT Conover Test	4w BT vs. End CT-RT Conover Test	2w vs. 4w after BT Conover Test
	p	p	p	p	p	p	p
***HDR***							
CD4^+^CD25^+^FoxP3^+^CD45RA^+^	< 0.0060	< 4.4E-05	< 0.0082	< 0.0109	< 0.0109	< 0.0082	< 0.7192
CD4^+^CD25^+^FoxP3^+^CD45RA^-^	< 0.0292	< 0.7521	< 0.3237	< 0.0034	< 0.4184	< 0.0035	< 0.0196
***PDR***							
CD4^+^CD25^+^Foxp3^+^CD45RA^+^	< 0.0955	< 0.0070	< 0.0020	< 0.0022	< 0.5959	< 0.6856	< 0.7767
CD4^+^CD25^+^FoxP3^+^CD45RA^-^	< 0.0384	< 0.0040	< 5.3E^-^05	< 0.0126	< 0.1075	< 0.5638	< 0.0389

**Fig. 2:**
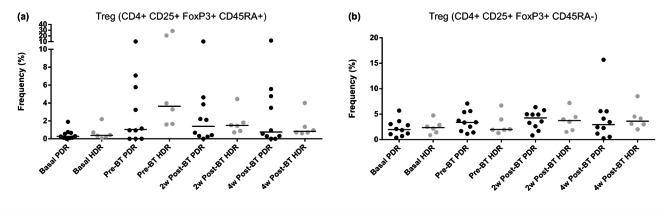
Comparison of median values of T regulatory cell populations during the study period at different time points between HDR and PDR brachytherapy groups: (**a**) Treg (CD4^+^CD25^+^FoxP3^+^CD45RA^+^) subpopulation; (**b**) Treg (CD4^+^CD25^+^FoxP3^+^CD45RA^−^) subpopulation. Significant changes are observed in the HDR group in both Tregs subpopulations at the different endpoints in Figure (**a**) y (**b**). In PDR patients, differences only are observed in CD45RA- subpopulation, Figure (**b**). However, no significant differences are shown in the percentage of Tregs between the HDR-BT and PDR-BT groups

No significant differences were found in the percentage of Tregs when HDR-BT and PDR-BT were compared using the Wilcoxon signed-rank test (Fig. 2).

### Myeloid-derived suppressor cells (MDSCs)

The strategy used to analyze different phenotypic and functional MDSCs subsets was defined as CD45 + CD33 + CD11b + cells. Then, MDSCs were also divided into subsets based on CD14 and HLA-DR expression.

Significant differences were observed in the MDSCs population (CD33 + CD11b+) when the different endpoints were compared in both groups of patients, HDR and PDR patients (Friedman one-way ANOVA p = 0.0052 for HDR and p = 7.864E-06 for the PDR ones).

The percentage of granulocytes (CD33 + CD11b + CD14-) did not show any significant change after the treatment in any comparison in the PDR group. However, a decrease in these cells was found at two weeks after BT treatment in HDR patients compared with the CRT endpoint (Conover test, p = 0.014).

For monocytes defined as (CD33 + CD11b + CD14 + HLADR−/low), a significant decrease in the percentage of these cells was observed after CRT treatment in both groups (Conover test, p = 0.012 in HDR patients and p = 0.005 in PDR patients). A summary of MDSCs values can be seen in Table 4, and Additional File.

**Table 4:** Difference of MDSCs subpopulation percentages between baseline and after completion of CRT and two and four weeks after completion HDR and PDR Brachytherapy

	Baseline vs. Treatment Friedman Test	CT-RT vs. Baseline Conover Test	2w BT vs. Baseline Conover Test	4w BT vs. Baseline Conover Test	2w BT vs. End CT-RT Conover Test	4w BT vs. End CT-RT Conover Test	2w vs. 4w after BT Conover Test
	p	p	p	p	p	p	p
***HDR***							
MDSC (CD33^+^CD11b^+^)	< 0.0051	< 0.0032	< 3.6E^-^05	< 0.0021	< 0.0274	< 0.7152	< 0.0470
G-CD33^+^CD11b^+^CD14^−^	< 0.0997	< 0.1074	< 0.1891	1	< 0.0140	< 0.1074	< 0.1891
Mo-CD33^+^CD11b^+^CD14^+ HLADR−/low^	< 0.0737	< 0.0120	< 0.0991	< 0.3788	< 0.2249	< 0.0433	< 0.2934
***PDR***							
MDSC (CD33^+^ CD11b^+^)	< 7.8E^-^06	< 1.0E-12	< 1.0E^-^12	< 3.0E^-^07	1	< 2.3E^-^05	< 2.3E^-^05
G-CD33^+^CD11b^+^CD14^-^	< 0.4580	< 0.8928	< 0.7090	< 0.1323	< 0.7090	< 0.1323	< 0.0874
Mo-CD33^+^CD11b^+^CD14^+ HLADR−/low^	< 0.2100	< 0.0057	< 0.0648	< 0.2065	< 0.2065	< 0.0648	< 0.4083

**Fig. 3:**
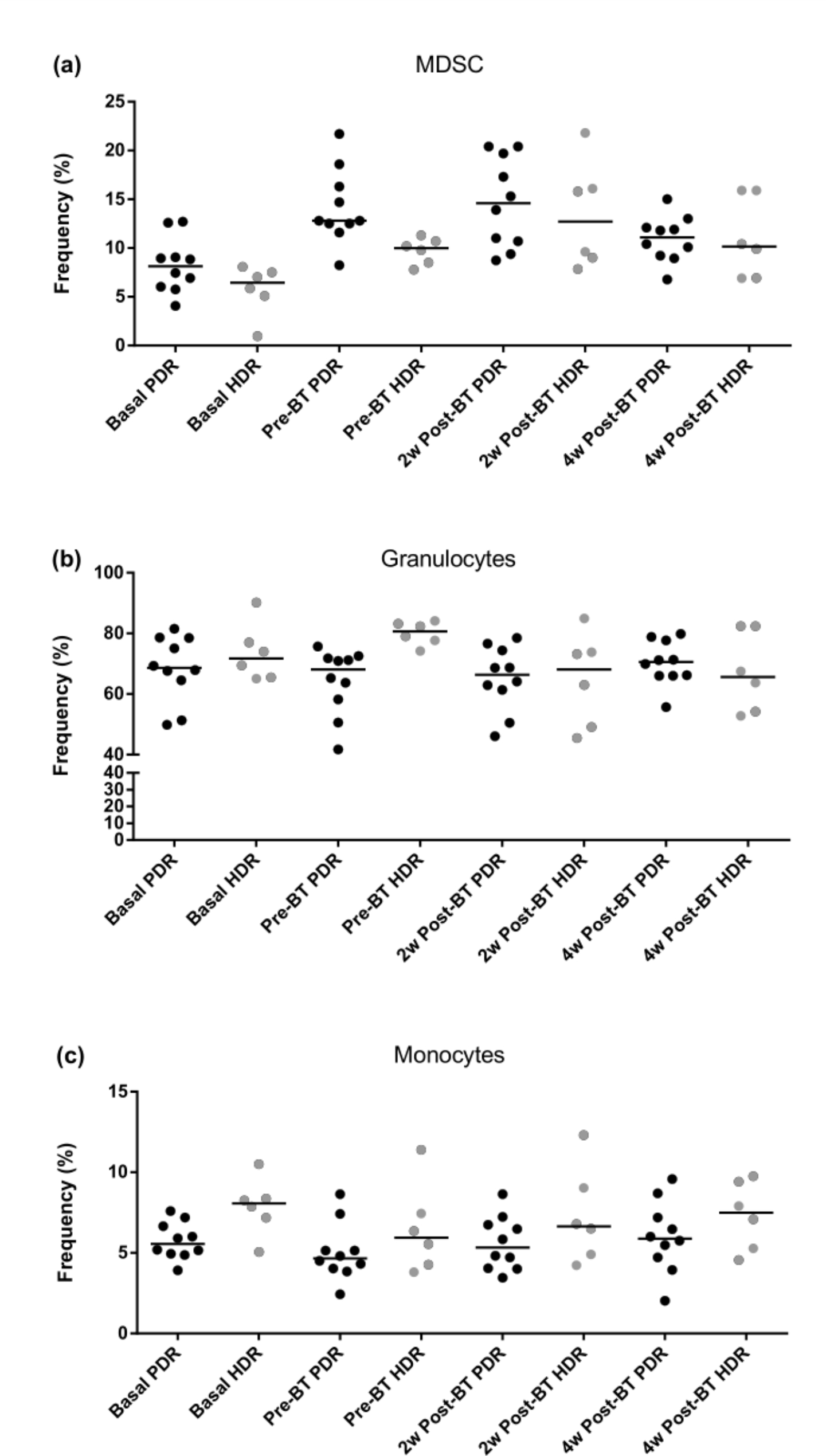
Comparison of median values of MDSC populations during the study period at different time points between HDR and PDR brachytherapy groups: (**a**) MDSC population; (**b**) Granulocytes subpopulation; (**c**) Monocytes subpopulation. No significant changes in the percentage of MDSCs, monocytes, and granulocytes between HDR-BT and PDR-BT are observed. However, a decrease in granulocytes is shown in Figure (**b**) at 2w post-BT treatment in HDR patients compared with the CRT endpoint. And a significant decline in monocytes after CRT treatment in both groups is shown in Figure (**c**)

No significant differences were found in the percentage of MDSCs, monocytes, and granulocytes when HDR-BT and PDR-BT were compared using the Wilcoxon test (Fig. 3).

## Discussion

In the present study, changes in the different cell populations of lymphocytes and immunosuppressor cells were observed in peripheral blood mononuclear cells after different radiation techniques, dose rates, and doses of radiation. When MDSCs and Tregs were compared with all lymphocytes, we observed that these two peripheral blood mononuclear cells (PBMCs) populations are more resistant to radiation and, consequently, dominate the cancer site after RT. Our results also reveal that NK cells are the best target to enhance immune responses in cervical cancer patients since these cell populations showed the highest treatment impact. The different cell populations along the treatment were diverse in the two treatment schemes compared in the present study (HDR-BT and PDR-BT). Better results were found in HDR-BT by an increase of CD56dimCD16 + NK cells.

The immune system cells are among the most highly radiosensitive cells [23]. As a result, transent lymphopenia occurs after radiation treatment [3, 4]. However, the radiosensitivity of lymphocyte populations is diverse. Although changes in the different lymphocyte populations were observed along with the treatment, when CD45 + lymphocytes were considered, these differences were not statistically significant. The lymphocytes subset with a higher impact on cell depletion was B lymphocytes. The lowest values were observed in this subset two weeks after BT treatment, while the cell recovery occurred four weeks after BT treatment.

Nevertheless, an increase was observed for the T lymphocytes subset and in the CD4+/CD8 + ratio after BT in the two groups (HDR and PDR). This result reinforces the use of BT for cervical cancer treatment since, in previous studies, a direct association between low CD4+/CD8 + ratios and poor cancer prognostic was observed [24, 25]. Increased CD4+/CD8 + ratio was caused by increased T lymphocytes CD4 + along with the treatment, while T lymphocytes CD8 + decreased up to 4 w after treatment when the recovery started. In initiating and maintaining immune response against cancer, CD4 + T lymphocytes play a crucial role. They are also involved in the antigen-specific response, where CD4 + cells help CD8 + develop memory cells [25, 26].

NK cells are essential in the cellular immune response to cervical cancer [27, 28]. In patients with positive cellular immune reactions to HPV-16 E6 and E7, regression of HPV-induced cervical intraepithelial neoplasia occurred [29, 30]. In addition, studies have indicated that NK cells can have an important role in metastasis reduction [31]. The present study observed that NK cells were the most sensitive to radiation treatment, showing different behaviors between cell subsets and PDR and HDR techniques. NK cells are characterized by two main functions, cytotoxicity and the production of interferon γ (IFN-𝛾). Both are differentially distributed with the different blood and lymphatic node cell subsets [32].

Depletion of NK cell activity is a common side effect of CRT [27]. We observed a decrease in the CD56dimCD16 + subpopulation after CRT treatment. This subpopulation is the one with the highest cytotoxic capacity [28, 32]. On the contrary, we observed an increase in the CD56dimCD16- NK subpopulation, with a less cytotoxic activity but an antitumor activity related to the secretion of cytokines, chemokines, and growth factors [33]. Also, an increased CD56-CD16 + NK subpopulation characterized by functional alterations due to their lower cytotoxic activity and cytokine production [32] was observed.

Promising results were obtained when the behavior of these cell subsets was evaluated when high doses per fraction with BT were administered. A higher percentage of CD56dimCD16 + cells was observed in patients who received HDR, especially those with increased expression of CD56highCD16+. This increase was evident even four weeks after the end of treatment. However, this was not observed in the PDR group of patients, where CD56dimCD16 + NK cells decreased along with the follow-up. The percentage of the NK56high cells increased, but it was not maintained. In contrast to the HDR group, at four weeks, the percentage of these cells began to decline. Therefore, we observed that patients who received treatment with HDR-BT achieved significantly higher values of these cells with a greater cytotoxic capacity [28, 32] than the PDR group, and the percentages were maintained throughout the study.

The subpopulation related to the secretion of immunoregulatory cytokines (CD56dimCD16- NK) had elevated values after CRT treatment. Lower values than baseline were observed in patients who received HDR, while the highest values of these cells were obtained two weeks after PDR-BT administration. These percentages decreased in this group of patients at four weeks, but they were higher than the baselines. However, these trends between the two BT treatment groups were not significant when both treatment arms were compared. Finally, the subpopulation of CD56-CD16 + NK cells known as dysfunctional [32] had higher percentages than baseline four weeks after completing BT in both groups. However, while a gradual decrease was observed in the HDR-BT group two weeks after BT, a progressive increase occurred in the other group. When both BT treatment techniques were compared, patients treated with PDR-BT increased the percentage of this subset of more inoperative cells, even one month after treatment.

When we studied the immunosuppressive component, the Tregs and MDSCs were the most radio-resistant subsets; this is in line with other published studies [23, 34, 35]. We observed an increase in the two immunosuppressive cell populations when CRT treatment was administered. Several studies have shown that the presence of Tregs suppresses the proliferation of effector T lymphocytes (CD8+) and negatively influences the prognosis of certain types of cancer. In addition, the loss of the CD45RA marker expressed by naive T cells due to the activation of the cells has been related to a worse clinical situation in patients since it limits the ability of the immune system to create an effective response against tumor cells [36]. RT allows the development of Tregs [34], but we detected that the behavior of these cells was different for patients who received the two treatment modalities for BT. We observed a decrease in the percentage of naive CD4 + CD25 + FoxP3 + CD45RA + Tregs after HDR-BT treatment, while an increase in activated CD4 + CD25 + FoxP3 + CD45RA- Tregs reached the maximum four weeks after BT. However, we observed an increase in the percentage of naive and activated cells in the patients receiving PDR-BT. These subpopulations started to decrease four weeks after BT administration. Although no significant improvement was evidenced for any of the two BT modalities, knowing the variations of these cells along the treatment makes the combination of immunotherapy easier with the different BT modalities used. For example, in the study presented here, patients who received PDR treatment could significantly benefit if an anti-Treg drug was administered within two weeks of completing the BT treatment.

On the other hand, studies have reported that abnormal accumulation of MDSCs in peripheral blood and tumors is an important immunological mechanism of T-cell anergy [7, 8, 37]. In fact, in patients with cervical cancer, the presence of these cells is associated with tumor burden and recurrence in the early and advanced stages of the disease [7]. Furthermore, irradiated tumors have previously been shown to recruit large bone marrow-derived myeloid suppressor cells [38]. Our study showed that the BT treatment and the EBRT did not reduce MDSCs percentages but increased up to four weeks after completing the treatment, that a decrease was observed in both groups. Furthermore, after the administration of CRT, the percentage of monocytes decreased in both groups, while the granulocytes showed resistance to EBRT. The percentage of monocytes and granulocytes after treatment did not change in those patients who received PDR-BT treatment. However, four weeks after the administration, the percentage of monocytes increased in patients who received HDR-BT, while a decrease in granulocytes was observed at two weeks of BT. These results indicate that both normofractionated and hypofractioned doses increased MDSCs. These data suggest that targeting MDSCs may increase antitumor immunity and increase the efficacy of therapies in cervical cancer patients.

## Conclusions

No previous studies have evaluated the effect of BT on peripheral immune cells in cervical cancer patients. As RT is a critical therapeutic approach for cervical cancer, it is an ideal model to investigate the effects of RT. We detected that NK cells are the best target to enhance immune responses in patients with cervical cancer since they were the most sensitive to BT treatment. We observed that patients who received treatment with HDR-BT achieved significantly higher values and for a longer time of the CD56dimCD16 + NK cells with greater cytotoxic capacity than the PDR-BT group, which presented their highest elevation in CD56-CD16 + NK cells. Compared to all lymphocytes (CD45+), a higher proportion of activated CD4 + CD25 + FoxP3 + CD45RA- Tregs are related to a worse clinical prognosis. However, high doses of radiation administered by HDR-BT modality reduced the percentage of the subpopulation of CD4 + CD25 + FoxP3 + CD45RA + Tregs but without being significant between both BT modalities. The information provided by this study can provide valuable predictive data. However, more studies with more significant numbers of patients are needed to support the HDR-BT modality to the detriment of PDR and combine BT with immunotherapy as a new therapeutic option in patients with cervical cancer.

### Electronic supplementary material

Below is the link to the electronic supplementary material.

**Additional File 1:** Changes in the different immune cell populations between baseline and after completion of CRT and two and four weeks after completion of HDR and PDR Brachytherapy

## Data Availability

All data generated or analysed during this study are included in this published article.

## List of abbreviations

BT: Brachytherapy
HDR: High-dose-rate
PDR: Pulsed-dose-rate
CRT: chemoradiation
NKs: Natural killer cells
MDSCs: Myeloid-derived suppressor cells
Tregs: Regulatory T cells
HPV: Human papillomavirus
CTLA-4: Cytotoxic T-lymphocyte antigen-4
PD-1/PD-L1: Programmed cell death-1/programmed cell death-ligand 1
EBRT: External beam radiation therapy
RT: Radiotherapy
SBRT: Stereotactic body radiation therapy
IORT: Intraoperative radiation therapy
EDTA: Ethylenediaminetetraacetic acid
FDR: False discovery rate
PBMCs: Peripheral blood mononuclear cells
IFN-𝛾: Interferónγ
